# Elevated levels of IL-12/IL-23p40 in Nova Scotia Duck Tolling Retrievers with autoimmune disease and lymphoma

**DOI:** 10.1038/s41598-024-62265-y

**Published:** 2024-05-21

**Authors:** Malin Nilsson, Sergey V. Kozyrev, Sara Saellström, Siri Johansson, Göran Andersson, Kerstin Lindblad-Toh, Helene Hansson-Hamlin, Henrik Rönnberg

**Affiliations:** 1 Department of Clinical Sciences, Swedish University of Agricultural Sciences, Uppsala, Sweden; 2 Department of Medical Biochemistry and Microbiology, Uppsala University, Uppsala, Sweden; 3 SciLifeLab, Uppsala University, Uppsala, Sweden; 4 Department of Animal Biosciences, Swedish University of Agricultural Sciences, Uppsala, Sweden; 5 Broad Institute of MIT and Harvard, Cambridge, MA USA; 6 Present Address: Anicura Kalmarsund Animal Hospital, Kalmar, Sweden

**Keywords:** Autoimmune, Immune-mediated rheumatic disease, Steroid-responsive meningitis-arteritis, IMRD, SRMA, NSDTR, Canine tumors, Cytokines, Cancer, Immunology

## Abstract

The Nova Scotia Duck Tolling Retriever (NSDTR) is predisposed to immune mediated rheumatic disease (IMRD), steroid-responsive meningitis-arteritis (SRMA) and certain forms of cancer. Cytokines are the main regulators of the immune system. Interleukin 2 is a cytokine involved in activation of T regulatory cells, playing a role in central tolerance and tumor immunity. Interleukin 12 and interleukin 23 share the same subunit, p40, and are both pro-inflammatory cytokines. The aim of this study was to compare levels of IL-2 in healthy NSDTRs to those with cancer or autoimmune disease and to compare levels of IL-12/IL-23p40 in healthy NSDTRs and beagles versus NSDTRs with cancer or autoimmune disease. 62 dogs were included in the analysis of IL-12/IL-23p40; healthy NSDTRs (n = 16), healthy beagles (n = 16), NSDTRs autoimmune (n = 18) and NDSTRs lymphoma/mastocytoma (n = 12) and 68 dogs for IL-2; healthy (n = 20), autoimmune (n = 36) and lymphoma/mastocytoma/adenocarcinoma (n = 12). NSDTRs with autoimmune disease had higher levels of IL-12/IL-23p40 compared to healthy dogs (p = 0.008). NSDTRs with lymphoma also had higher levels of IL-12/IL-23p40 compared to healthy NSDTRs (p = 0.002). There was no difference in levels of IL-2 between healthy and diseased NSDTR. Statistical analysis was performed using Bonferroni corrections for multiple testing. These findings can contribute to the knowledge of autoimmune disease and cancer in dogs.

## Introduction

The Nova Scotia Duck Tolling Retriever (NSDTR) is a dog breed originating from Canada. Several reports have shown an increased risk for immune mediated diseases in the breed^1–5^. Two of the immune-mediated diseases found most commonly in NSDTRs are immune-mediated rheumatic disease (IMRD) and steroid-responsive meningitis-arteritis (SRMA)^1–5^. IMRD is a non-erosive arthritis with affected dogs suffering from joint pain, stiff gait and sometimes, other signs such as fever and pain from the musculature upon palpation^1^. Clinical signs are in most cases, waxing and waning. The disease is often referred to as SLE-related as it resembles the autoimmune disease systemic lupus erythematosus (SLE). Both SLE patients and dogs with IMRD have non-erosive arthritis affecting multiple joints^1,6^. NSDTRs with IMRD sometimes have skin lesions just like humans with SLE and the majority have circulating antinuclear antibodies (ANA), as do humans with SLE^1,6,7^. Individuals diagnosed with SLE, both humans and dogs, commonly exhibit hematological changes such as anemia or thrombocytopenia, which are not normally observed in NSDTRs with IMRD^1,6,8,9^. For humans, there is a list of criteria to be fulfilled before diagnosing SLE, similar criteria have been proposed for canines but they are not well established^7,9^. Both in humans and dogs, there are diseases with similar clinical signs as SLE but that do not fulfill the criteria for SLE, those are referred to as SLE-related disease^10–13^. SRMA is a noninfectious meningitis that typically results in acute neck pain, fever and stiff gait^14^. SRMA cases are in general ANA negative^2^. NSDTRs have an increased risk for immune-mediated diseases and they are also overrepresented for certain forms of cancer^15^. Tumors commonly seen in NSDTRs are, for example, mast cell tumors and lymphomas^3,15^. Humans with SLE are known to have an increased risk for lymphoma^16,17^.

Previous studies have identified eleven genes associated with increased risk for developing SRMA and IMRD in NSDTRs, one of which is shared between the diseases^18^. Several of the genes found are involved in T cell activation via the nuclear factor of activated T cells (NFAT) pathway^19^. The NFAT pathway was first described as an activator of IL-2 transcription but is also involved in several other processes^20,21^. Another risk factor for developing IMRD in NSDTRs is a particular risk haplotype of MHC class II^22^.

Cytokines are major regulators of the immune system and the inflammatory responses, and thereby important players in autoimmune diseases. A study from 2018 by Bremer et al.^23^ identified interleukin enhancer binding-factor 2 and 3 (ILF-2 and ILF-3) as antigens targeted by autoantibodies in NSDTRs with IMRD. ILF-2 is one of the transcription factors required for expression of the *IL-2* gene^24,25^. In one study, cell cultures from human individuals with SLE were found to produce less IL-2 than cells from healthy individuals when stimulated and in a murine model, deficiency of the IL-2 receptor lead to development of hemolytic anemia with autoantibodies and death occurring at 12 weeks of age^26,27^. Other murine models of SLE have also shown a defect production of, and response to IL-2^28,29^. IL-2 is a key cytokine in T cell activation; it is secreted primarily by activated CD4^+^ T-helper cells, and stimulates cell proliferation, antibody and cytokine production and cytotoxicity. IL-2 also has a role in the maintenance of peripheral tolerance through its role in the development and survival of regulatory T cells (Treg)^30–34^. Tolerance to self-antigens is an essential function of the immune system, and loss of tolerance or lack of establishment of tolerance can both lead to development of autoimmune diseases. Central tolerance is established early in life in the thymus where autoreactive lymphocytes are eliminated by negative selection through apoptosis. However, some autoreactive lymphocytes will escape the selection in the thymus and have potential to cause autoimmune disease. The peripheral tolerance is responsible for eliminating these autoreactive cells, and Treg cells are crucial in this process^31,35^.

Treg cells play a vital role not only in the establishment and maintenance of tolerance but also in tumor immunity, where they can promote progression of malignancies by suppressing the immune response, thus contributing to the tumor immune-evasion^36,37^. This is considered one of the fundamental hallmarks of cancer development^38^. During the last decade a new field of cancer therapy, immune checkpoint inhibitors have been developed based on this knowledge. Immune checkpoint inhibitors are based on the knowledge that the T cell response can be enhanced by blocking the inhibitory mechanisms of the immune system, which may lead to a more effective rejection of tumor cells^39^. Moreover, many cancers are developed out of the chronic inflammatory state, stressing the importance of tumors trying to initiate and keep this type of inflammatory status^40^.

**Figure 1:**
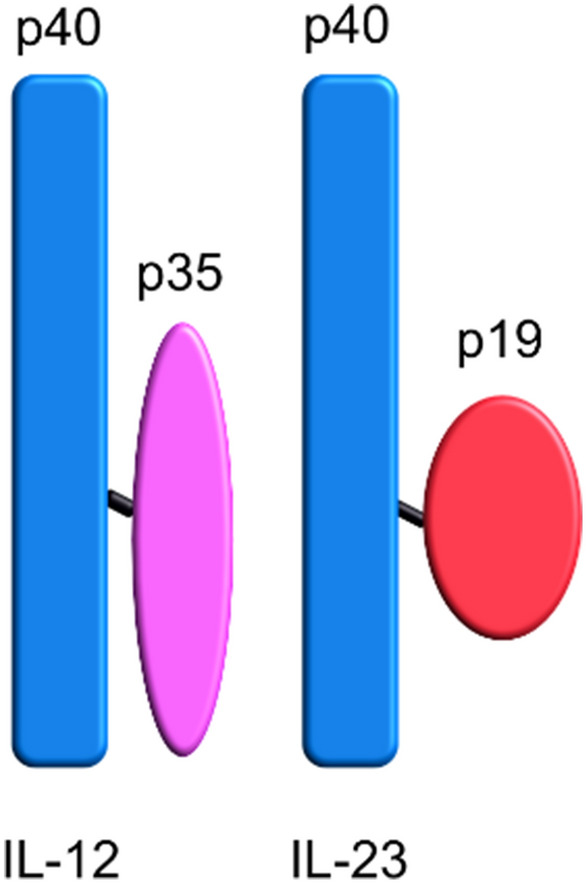
Schematic picture of IL-12 and IL-23. IL-12 consist of the p35 and p40 subunits, IL-23 consist of the p40 and p19 subunits. Picture showing the p40 subunit in blue. The two other subunits, p35 and p19 shown in purple and red.

Interleukin 12 (IL-12) and interleukin 23 (IL-23) are both members of the IL-12 cytokine family, a family unique in having heterodimeric cytokines. They both consist of two covalently linked subunits; IL-12 of the heavier p40 subunit and the lighter p35 subunit and IL-23 is composed of the same p40 unit as IL-12 but dimerized to p19 instead of p35 (Fig. 1). IL-12 is secreted by antigen presenting cells (APCs) upon activation and has pro-inflammatory actions. The major effect of IL-12 is to induce T lymphocytes to differentiate into T helper 1 cells (Th1) that produces interferon gamma (IFN-γ)^41^. Dendritic cells and macrophages produce IL-23 and although IL-23 has pro-inflammatory properties, just as IL-12, they do not have the same function. IL-23 promotes a T cell response characterized by interleukin 17 (IL-17) production. In human SLE there are studies suggesting that there is an imbalance between Th1 and Th17 as well as Treg and Th17 response in patients compared to controls^42,43^. In canines with osteosarcoma, a previous study has shown increased levels of IL-12/IL-23p40 in serum^44^. NSDTR dogs are at high risk for developing autoimmune disease as well as cancer compared to other breeds, however, the cause of this increased risk remains incompletely known. Therefore, the aim of this study was to compare levels of IL-2 and the p40 subunit of IL-12 and IL-23 (IL-12/IL-23p40) in blood from healthy NSDTRs and NSDTRS with a diagnosis of IMRD, SRMA or cancer. A second aim was to compare levels of IL-12/IL-23p40 from healthy NSDTRs with healthy beagles, another canine breed not prone to develop either IMRD or cancer.

## Material and methods

### Ethical approval

This study is reported in accordance with ARRIVE guidelines. All animal experiments complied with national legislation and had ethical approval from the Swedish Board of Agriculture—ref number Dnr C15/16 and 5.8.18-00065/2021.

### Sampling and population

All included NSDTRs were privately owned and all the dog owners signed a written consent for each dog that participated in this study. All included beagles were teaching dogs, owned and housed at the Swedish University of Agricultural Sciences. All of the NSDTRs included in the healthy group, were considered healthy by their owners and by a brief clinical examination at the time of blood sampling. The beagles regularly go through clinical examinations by veterinarians and students and they were considered healthy by their caretaker. None of the healthy dogs had any suspected or diagnosed chronic disease and had not suffered from any acute disease or trauma during the last week before sampling, and none of them received any medication. All healthy NSDTRs, except one, were sampled between spring 2020 and summer 2021. One dog was sampled by a local veterinarian in February 2016. The beagles included were sampled in the autumn of 2020 and during the spring of 2023.

The dogs considered to be affected by IMRD had a diagnosis based on a mix of medical history, physical examination, an indirect immunofluorescence ANA (IIF-ANA) test, arthrocentesis and radiographs. Blood samples were collected between 2017 and 2021.

The dogs with tumors all had a diagnosis of cancer based on findings during physical examination, diagnostic imaging, blood samples, cytology or histopathology.

The dogs with SRMA had a diagnosis based on a mix of medical history, physical examination, blood samples and analysis of cerebrospinal fluid (CSF). For all of the diseased dogs, IMRD, cancer and SRMA, the diagnostic work up was at the discretion of the treating veterinarian, therefore it differed from case to case.

Blood samples from the beagles and the healthy NSDTRs were drawn from the cephalic vein using a vacutainer system. Blood was collected in serum tubes and left to clot for a minimum of 30 min, and then centrifuged at 3000 rpm for 10 min. The serum was aspirated into cryo-tubes and frozen in – 80 °C. In some cases, the cryo-tubes were kept at + 4 °C for 1–5 days before freezing due to logistical reasons. Blood samples from the NSDTRs with IMRD, SRMA or cancer, were either collected by a veterinarian within the research group (in the same way as described above) or sampled by local veterinarians, from different veterinary clinics throughout Sweden, according to the routines at the contributing clinics. Samples sent in by other veterinarians were first frozen at − 20 °C and later transferred to − 80 °C for long-term storage.

### IL-12/IL-23p40

In total 46 NSDTRs and 16 beagles were included in the analysis of IL-12/IL-23p40 in serum. The NSDTR dogs were divided into four different groups: healthy (n = 16), IMRD (n = 18) lymphoma (n = 5) and mast cell tumors (n = 7). All of the beagles were healthy (n = 16). All of the healthy NSDTRs and all of the NSDTRs with IMRD used in the analysis of IL-12/IL-23p40 were also included in the analysis of IL-2. None of the beagles were included in analysis of IL-2. Except one dog, all of the dogs with cancer used in the analysis of IL-12/IL-23p40 were also included in the analysis of IL-2. All information about breed, sex, age, diagnosis, treatment, and ANA reactivity for dogs is included in supplementary information 1.

All samples had been frozen and stored in – 80 °C until analysis. Number of years in the freezer, ranged from zero to six with a median of one year.

### IL-2

In total, 68 NSDTRs were included in the analysis of IL-2 levels in serum. The NSDTRs were divided into four different groups: healthy (n = 20), IMRD (n = 27), cancer (n = 12) and SRMA (n = 9). All information about breed, sex, age, diagnosis, treatment, and ANA reactivity for dogs is included in supplementary information 1.

As for IL-12/IL-23p40 all samples were stored in – 80 °C. Numbers of years in the freezer ranged from zero to seven with a median of one year.

### ELISA analysis

Two commercially available enzyme linked immunosorbent assays (ELISA, *RayBio*® *Canine IL-2 ELISA Kit* and *RayBio*® Canine IL-12p40 ELISA Kit*, Atlanta, GA, USA*) were used to quantify levels of IL-2 and IL-12/IL-23p40 in serum. The samples were prepared according to the manufacturer’s instructions. All samples were thawed at room temperature and were analyzed as triplicates in 96 well plates. The value reported for each dog is the average of the measured values.

All serum samples were diluted 1:2 for IL-2 analysis and 1:3 for IL-12/IL-23 p40. Supplied standard proteins were used to prepare a standard solution of 100 ng/ml for IL-2 and 50 ng/ml for IL-12/IL-23p40. A 2:5 dilution series in seven steps were performed for both cytokines. Detection antibodies for IL-2 and IL-12/IL-23p40 and Horseradish Peroxidase (HRP)-Streptavidin were prepared shortly before use.

For both assays, 100 µl of each sample and a standard were added and incubated at room temperature for 2.5 h. After a washing procedure with *Wellwash™ Microplate Washer* (*Thermo Scientific Inc,* Life Technologies BV/Thermo Fisher Scientific, Stockholm, Sweden), 100 µl of biotinylated antibody was added to each well and incubated with gentle shaking at room temperature for 1 h. After a new washing procedure, 100 µl of HRP-Streptavidin solution was added to each well, and the plates were then incubated at room temperature for 45 min. The washing procedure was then repeated and thereafter, 100 µl of tetramethylbenzidine (TMB) One-Step Substrate Reagent was added to each well before plates were incubated in darkness at room temperature for 30 min. Stop Solution was added and intensity was measured at 450 nm using a *Multiskan EX* spectrophotometer (*Thermo Scientific Inc,* Life Technologies BV/Thermo Fisher Scientific) and *Ascent Software*.

### Intra-assay variation

The intra-assay coefficient of variation (CV) was recommended from the manufacturer to be below 10% for both of the assays.

In the IL-12/Il-23p40 ELISA, the intra-assay CV was less than 10%. The average CV of all three plates were 6.8, 6.1 and 5.5%, respectively. There were nine samples with a CV higher than 10%. The CV of these samples ranged between 10.7 and 22.1%. The samples with a CV-% higher than ten had a low absorbance and were therefore not excluded from analysis.

In the IL-2 ELISA, the correlation coefficient of the three plates were 0.96, 0.99, and 0.97, respectively. The intra-assay CV for the three plates were 9.8, 5.8 and 8.5%. There were 16 samples with a CV higher than 10%. The CV of these samples ranged between 10.4 and 23.3%. The samples with a CV-% higher than ten all had a low absorbance and were therefore not excluded from the analysis.

### Data analysis

For both assays, if values were above or below the range of the assay, we choose to use the lowest standard value for values lower than measurable and the highest standard value for values higher than measurable (min/max range from 28.58 to 6000 pg/ml for IL-12/IL-23p40 and from 0.41 to 100 ng/ml for IL-2). For statistical analysis, a commercially available software program (JMP Pro v. 17.2.0, Cary, NC, USA) was used. Since the data was not normally distributed, median and interquartile range (IQR) is presented. Data was analyzed using descriptive statistics as well as with the nonparametric Wilcoxon rank sum test. Statistical significance levels were set at 0.05. To correct for type one errors when multiple groups were compared we used Bonferroni correction. For IL-12/IL-23 p40 two different calculations were made, one with two comparison and another with four groups (healthy, IMRD, lymphoma and MCT) giving six comparisons. For IL-2 we tested four groups (healthy, IMRD, SRMA and cancers) giving eight different comparisons and p-values were adjusted accordingly.

## Results

### IL-12/IL-23p40

Sex and age distribution of dogs included in the analysis of IL-12/IL-23p40 are shown in Table 1. For additional information about each dog, see Supplemental Table 1.

**Table 1:** The distribution of age and sex among dogs included in ELISA analysis of IL-12/IL-23p40 in serum.

	NSDTR healthy	NSDTR IMRD	NSDTR lymphoma	NSDTR mast cell tumor	Beagles healthy
Total number (n)	16	18	5	7	16
Males	7	8	2	2	4
Females	8	9	2	3	10
Neutered males	1	1	0	2	2
Neutered females	0	0	1	0	0
Age average in years	5.4	5.1	10.4	7.7	5.3
1–3 years	4	5	0	0	6
4–6 years	6	8	0	2	7
7–9 years	6	6	2	4	2
10 years or older	0	0	3	1	1

Number of dogs in each category.

None of the healthy dogs received any medications. In the IMRD group, six of the dogs were untreated, seven dogs received corticosteroids in varying doses, four dogs received non-steroidal anti-inflammatory drugs (NSAID), and one dog received ursodeoxycholic acid due to concurrent liver disease. In the IMRD group, 15 dogs were positive for IIF ANA, two were negative, and for one dog, data about ANA reactivity was missing.

All lymphoma samples used in this study were from naïve cases, prior to any anti-cancer treatment. Not all dogs with mast cell tumors were naïve; three out of seven dogs had received treatment prior to blood sampling. One dog was treated with corticosteroids, one had the mast cell tumor surgically removed with unclean margins in all directions and had undergone radiation therapy before sampling, and the last one had the tumor surgically removed with signs of metastasis and was therefore treated with corticosteroids and vincristine after surgery.

In the group of healthy NSDTRs, one dog had an extreme IL-12/IL-23p40 value of 5518 pg/ml and was therefore excluded from further analysis. Among the remaining NSDTR dogs, the levels of IL-12/IL-23p40 ranged from 71 to 787 pg/ml. The median for all healthy NSDTRs were 288 pg/ml (IQR 134–340). For the NSDTRs with IMRD, the level of IL-12/IL-23p40 ranged from 75 to 1581 pg/ml (median 604 pg/ml IQR 318–997).

**Figure 2:**
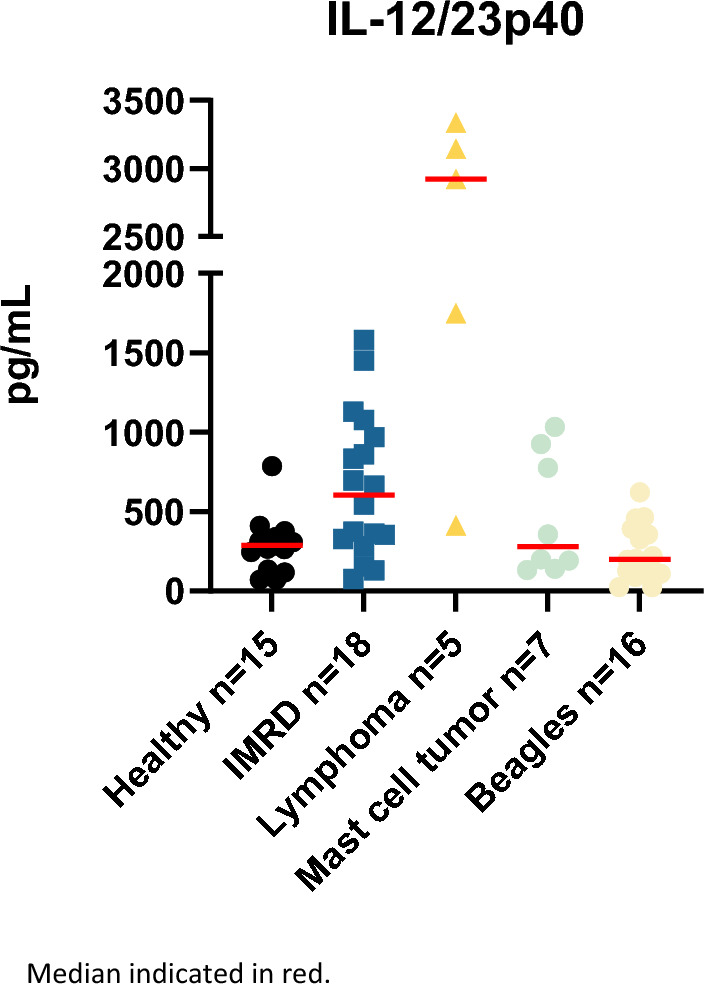
Levels of IL-12/IL-23 p40 for the five groups, healthy NSDTRs, NSDTRs with IMRD, NSDTRs with lymphoma and mast cell tumors and healthy beagles. Median indicated in red.

There were five dogs with lymphoma and seven dogs with mast cell tumors, both cutaneous and subcutaneous, of different grades (suppl. Table 1). The IL-12/IL-23p40 levels of the dogs with cancer ranged from 133 to 3335 pg/ml (median 851 pg/ml IQR 195–2627). For the beagles, one of the dogs had a value lower than the assay could detect and another beagle had a value lower than what should have been detected, for those dogs the value was set to the lowest detectable value on the standard curve (24.6 pg/ml). These values were included when median and IQR were calculated. All data points are shown in Fig. 2. For the healthy beagles, the values ranged from 25 to 622 pg/ml (median 199 pg/ml IQR 109 to 383).

When comparing the different groups (healthy, IMRD, mast cell tumors and lymphoma), there was a statistically significant difference between healthy NSDTRs and NSDTRs with IMRD (p = 0.008). There was also a statistically significant difference between healthy NSDTRs and NSDTRs with lymphoma (p = 0.002) but not for healthy dogs and dogs with mast cell tumors.

When considering absolute numbers, four out of five dogs with lymphoma had values of higher than 1500 pg/ml, only one dog out of seven with mast cell tumor had a value higher than 1000 pg/ml (1032 pg/ml). Median for dogs with lymphoma was 2920 pg/ml (range 414–3335 pg/ml) and for dogs with mast cell tumors 200 pg/ml (range 133–1032 pg/ml) (Fig. 2).

**Figure 3:**
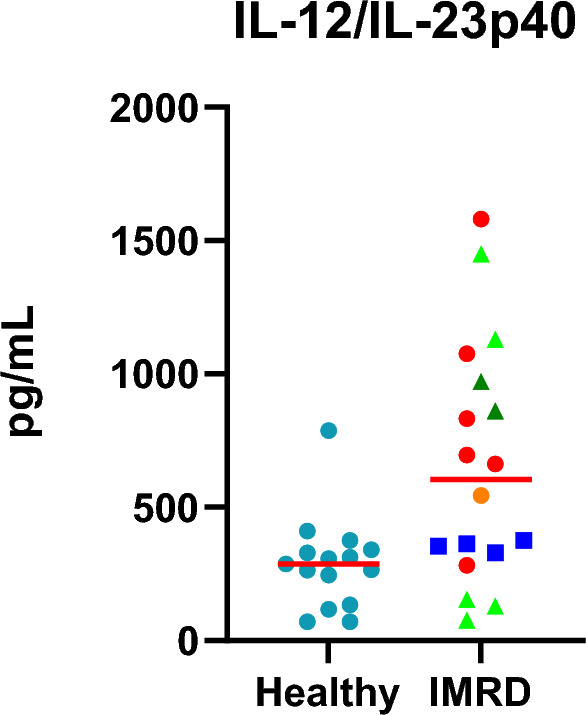
Levels of IL-12/IL-23p40 in healthy NSDTRs and NSDTRs with IMRD. None of the healthy dogs was treated. Dogs with IMRD were either untreated (red filled circles), treated with NSAID (blue filled squares) or treated with prednisolone low doses (less than 1 mg/kg) (light green filled triangles) or high dose prednisolone (1 mg/kg or more) (dark green filled triangles). Median indicated in red.

We observed a trend that dogs in the IMRD group treated with NSAID had a lower level of IL-12/IL-23p40 than dogs treated differently (Fig. 3).

Dogs treated with NSAID had a median value of 358 pg/ml (range 329–375 pg/ml) while dogs treated with corticosteroids, (both high and low dose) had a median of 861 pg/ml (75–1450 pg/ml). Since there were only four dogs in the group of dogs treated with NSAID, no further statistical calculations were made.

There was no difference in values between the healthy dogs of the two breeds (p = 0.33).

## IL-2

Age- and sex distribution of the dogs included in the analysis of IL-2 is presented in Table 2.

**Table 2:** Age and sex among dogs included in IL-2 analysis.

	Healthy	IMRD	Cancer	SRMA
Total number (n)	20	27	12	9
Males	8	10	4	7
Females	9	13	5	2
Neutered males	2	3	1	0
Neutered females	1	1	2	0
Age average in years	5.2	5.1	8.5	1.1
1–3 years	7	9	0	9
4–6 years	6	11	4	0
7–9 years	6	6	4	0
10 years or older	1	1	4	0

As previously described, none of the healthy dogs received any medications. In the IMRD group, 18 of the dogs were also included in the analysis of IL-12/IL-23p40 and described above. For the remaining nine dogs, five were untreated, two dogs received corticosteroids in varying doses and one dog received NSAID. For one dog, data about treatment was missing. In the IMRD group, 23 dogs were positive for ANA, three were negative and for one dog, data about ANA reactivity was unavailable.

In the cancer group, there were five dogs diagnosed with lymphoma and six dogs with mast cell tumors, both cutaneous and subcutaneous, of different grades and one dog with an adenocarcinoma in the small intestine. The dog that was diagnosed with adenocarcinoma in the small intestine did also suffer from IMRD and was included in the IMRD group as well but with a sample taken at a different time. The IMRD sample was taken three years before the dog received a diagnosis of cancer. At the time of the cancer diagnosis, the autoimmune disease was not in an active state. The dog with adenocarcinoma in the small intestine underwent surgery at diagnosis, however the tumor reoccurred. The sample was taken at reoccurrence before any anti-cancer treatment was given. The dogs with lymphoma and mast cell tumors were the same dogs as in the IL-12/IL-23p40 assay and described above.

In the SRMA group, two of the dogs were untreated, five dogs were treated with corticosteroids in varying doses. For all details about treatment see Supplementary Table 1.

In the group of healthy NSDTRs, one dog had an extreme IL-2 value of 46.2 ng/ml (see suppl. Table 1) and that dog was excluded from further analysis. This was not the same dog as the dog with an extreme value in the analysis of IL-12/IL-23p40. Among the remaining healthy dogs, the levels of IL-2 ranged from 0.4 to 8.0 ng/ml. Of the healthy dogs, five had levels of IL-2 below the detection limit, for those samples the value was set to the lowest detectable standard value (0.4 ng/ml). The median for all healthy NSDTRs were 1.53 ng/ml and IQR 0.41–2.0. One of the dogs with IMRD had a level of IL-2 greater than the assay could measure. For that sample, the value was set to the highest detectable value on the standard curve (100 ng/ml). There was one IMRD-affected dog with a level of IL-2 below the detection limit, also for this sample the value was set to the lowest detectable standard value (0.4 ng/ml). For the other NSDTRs with IMRD the level of IL-2 was 1.0–41.5 ng/ml. For all NSDTRs with IMRD the median was 1.9 ng/ml (IQR 1.6–5.8).

**Figure 4:**
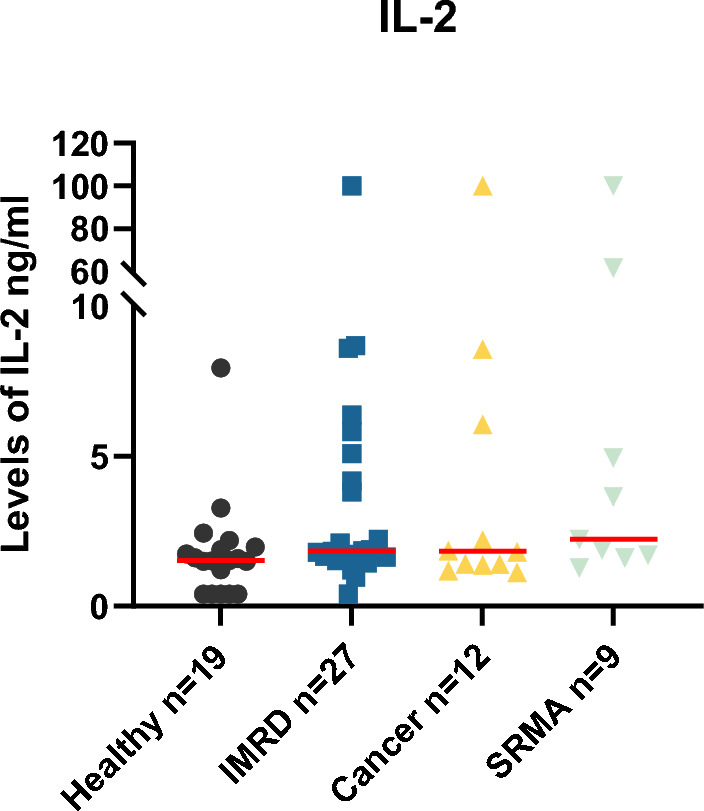
Levels of IL-2 the groups, healthy dogs, dogs with IMRD, dogs with SRMA and dogs with cancer. Median indicated in red.

One of the dogs diagnosed with lymphoma had a value greater than the assay could detect, for that sample the value was set to the highest detectable value on the standard curve (100 ng/ml). For the other NSDTRs with cancer the levels of IL-2 were 1.1–48.8 ng/ml. The median for all dogs diagnosed with cancer was 1.8 ng/ml and IQR 1.3–7.9. Among the NSDTRs with SRMA, there was one sample with level of IL-2 greater than the assay could measure, that sample was handled as previously and the value was set to the highest detectable value on the standard curve (100 ng/ml). For the remaining dogs, the IL-2 levels were determined to be 1.3–61.1 ng/ml. Median for all dogs with SRMA was 2.2 and IQR 1.7–5.0. All data points are shown in Fig. 4.

When comparing the different groups (healthy, IMRD, SRMA and cancer) significance was not reached.

## Discussion

This study aimed at comparing levels of the cytokines IL-12/IL-23p40 and IL-2 in serum samples obtained from healthy and diseased NSDTRs. We found that NSDTRs with IMRD and lymphoma had significantly higher levels of IL-12/IL-23p40 compared to healthy NSDTRs.

A study from Lauwerys et al.^45^ found that human SLE patients had higher IL-12/IL-23p40 levels in serum compared to healthy controls, that study concluded that it was the level of free p40 monomer that was elevated in patients. They also found that levels of p40 correlated to disease activity. Other studies of SLE patients have reported higher levels of IL-12 in the serum of patients, compared to healthy controls^42,46^. IL-23 promotes a T cell response characterized by interleukin 17 (IL-17) production by T helper 17 cells (Th17). These Th17 have been suggested to be involved in development of autoimmune inflammation^47–49^. Elevated levels of IL-23 were recently detected in human patients with SLE^50,51^. IMRD in NSDTRs and SLE in humans are similar in many ways; they both cause a chronic autoimmune inflammation leading to a non-erosive arthritis and both are generally waxing and waning. The majority of IMRD cases in NSDTRs are positive for ANA^1^. In human SLE, these antibodies are an immunological hallmark of disease. In our study, we observed that the level of IL-12/IL-23 p40 was higher in dogs with IMRD as compared with healthy dogs. If this is the free monomer, a homodimeric p40 or p40 in combination with p19 or p35, cannot be concluded from the analysis, due to the commercial assay design. However, considering that, this is a disease, which lacks a gold standard for stating the diagnosis; cytokines might be able to help in the diagnostic work up and potentially to identify the waxing versus waning stages of disease, no matter whether it is the free monomer or IL-12 or complete IL-23. To our knowledge, this is the first study measuring IL-12/IL-23p40 in dogs with autoimmune disease and lymphoma.

We observed that untreated dogs and dogs treated with a high dose of corticosteroid in general had a higher level of IL-12/IL-23p40 than dogs on a low dose of corticosteroids or NSAID. Higher doses of corticosteroids are generally given to dogs with more severe clinical signs and therefore likely a more active disease and the dose is normally tapered when clinical signs resolve. A possible explanation to why dogs given NSAIDs in general had a lower value is that NSAID to a larger extent is given to dogs less severely affected with disease than dogs given corticosteroids. However, we cannot exclude that this effect could be due to better suppression of inflammation by non-steroid anti-inflammatory treatment. In humans, autoimmune rheumatic diseases (AIRDs) comprise a diverse group of conditions characterized by numerous shared features. The initial therapy of these patients is often generalized immunosuppressive treatment, however, with a growing knowledge regarding the mechanisms behind disease new therapies have emerged^52^. Example of these new therapies are inhibition of different cytokines such as IL-6, IL-17 and TNF-alpha^53^. In recent decades, increasing evidence suggests that chronic inflammation can promote cancer^54–56^. Inflammation is a fundamental process for tissue repair and regeneration, and it also provides a microenvironment with factors essential for malignant transformation, tumor growth and metastasis. Several studies have reported an overall increase in risk for cancer in human SLE patients and for hematological malignancies in particular^57^. The NSDTR population in Sweden has been shown to have an increased risk for lymphoma in comparison with all other dog breeds^3^. The cause of this increased risk is not known, neither in dogs nor in humans. Comparative oncology is a growing field and dogs have proven useful as a comparative model of spontaneous diseases^58^. This could contribute to both increased animal welfare and quality of life for dogs as well as increasing the understanding of similar conditions in humans.

NSDTRs with lymphoma had a statistically significant different level of IL-12/IL-23p40 compared to healthy NSDTRs. There were only five dogs with lymphoma included in this study, which is a small number to draw definitive conclusions, but these findings encourage further studies. The role of both IL-12 and IL-23 in cancer have been debated as they have been shown to have both pro- and anti-tumor activities^59^. In several human cancers, IL-23 have been overexpressed compared to healthy controls^60–62^ and as previously mentioned there is one study that reports elevated IL-12/IL-23p40 levels in dogs with osteosarcoma^44^.

There was no difference between the healthy dogs from the two different breeds regarding levels of IL-12/IL-23p40. It is therefore likely that the changes we found between healthy and diseased dogs are because of disease.

We detected no significant differences in the values of IL-2 between healthy NSDTRs and NSDTRs with disease. There was however a large difference in values within the group of dogs with IMRD. This could be due to the fact that there were both treated and untreated dogs included in the study. Moreover, the treatment had been performed with different medications and with different doses. The group of dogs with IMRD is homogenous in terms of diagnosis, but not if their disease activity is considered. It is challenging to find dogs that are equal in disease activity and there are no objective measurements to assure this equality. In studies of humans with SLE, IL-2 levels have been found to be lower in patients with SLE compared with healthy controls, regardless of corticosteroid treatment^26^. This finding could not be confirmed in the dogs included in this study. However, Bremer et al.^23^ showed that NSDTRs with IMRD and a positive IIF-ANA of a speckled pattern in general carry autoantibodies against the transcription factors ILF-2 and ILF-3, crucial for transcriptional regulation of *IL-2* and *IL-13* gene expression. One could speculate that these autoantibodies could generate a different IL-2 response in NSDTRs with IMRD in comparison to humans with SLE. ILF2 autoantibodies are a rare finding in human autoimmune disease^63^.

The levels of IL-2 in serum among the dogs included in this study were generally low, for example, it was below the assay detection level, for five of the healthy dogs. Instructions for the assays were followed as stated from the manufacturer, for the IL-2 assay samples were diluted 1:2, in hindsight, it could have been better not to dilute the samples at all. Due to lack of serum, samples with a low absorbance could not be re-run.

The CV values were, in both of the assays, generally within preferred range. A number of samples had a slightly higher CV, but we accepted a higher CV when absorbance’s were low. When the absorbance is low, a very small difference in values between the replicates leads to a higher variation of coefficient. The average CV were in both assays well within the range of 10%.

Some samples were stored in refrigerator for a few days before freezing. We could not identify any abnormalities or adverse patterns of results in samples depending on handling after sampling.

### Limitations

There are limitations in this study. First, the autoimmune dogs included were at different stage of disease, which could possibly affect the levels of cytokines. However, in the study from Capper et al.^46^ they found IL-12 to be elevated in both active and inactive human SLE-patients in comparison to healthy controls. Another limitation is that there are both treated and untreated dogs included in the study. Larger groups of dogs treated in the same manner would be optimal, however, given the relatively low prevalence of disease this is challenging to achieve. Moreover, with the current ELISA assay, it cannot be concluded whether or not the p40 measured derived from IL-12 or IL-23 or as the free monomer. Further studies are needed to investigate this matter.

## Conclusion

In conclusion, we detected elevated levels of IL-12/IL-23 p40 in serum for NSDTRs with immune-mediated disease compared to healthy controls. We also detected higher levels of IL-12/IL-23p40 in serum from NSDTRs with lymphoma compared to healthy NSDTRs. These findings need further studies for confirmation, in a larger cohort of dogs of different breeds to take into account genetic differences contributing to susceptibility to autoimmune diseases and cancer in dogs. However, these findings could be one piece of the puzzle of the interplay between autoimmune disease and cancer in dogs and contribute to our understanding of similar conditions in humans.

### Supplementary Information

Supplementary Table 1.

## Data Availability

The data are not publicly available since they contain information that could compromise research participant privacy, but are available from the corresponding author upon reasonable request.

## Abbreviations

ANA: Antinuclear antibodies
CV: Coefficient of variation
ELISA: Enzyme linked immunosorbent assay
IL-2: Interleukin 2
IL-12: Interleukin 12
IL-23: Interleukin 23
IMRD: Immune mediated rheumatic disease
NFAT: Nuclear factor of activated T cells
NSAID: Non steroid anti-inflammatory drugs
NSDTR: Nova Scotia Duck Tolling Retriever
SLE: Systemic lupus erythematosus
SRMA: Steroid-responsive meningitis-arteritis
Treg: T regulatory cells
