# Supplementary material for: Elevated levels of IL-12/IL-23p40 in Nova Scotia Duck Tolling Retrievers with autoimmune disease and lymphoma
Source: Sci Rep. 2024 May 21;14:11624. doi: 10.1038/s41598-024-62265-y (PMC11109178; doi:10.1038/s41598-024-62265-y)
Supplement: Supplementary file 1 — Supplementary Table 1. [file 41598_2024_62265_MOESM1_ESM.docx]

Information about breed, sex, age, diagnosis, treatment and ANA reactivity for dogs included in the analysis of IL-2 and/or IL-12/IL-23p40 in serum.

| Dog number | Breed | Sex | Age at sampling, years | Diagnosis | Treament | ANA | Included in IL12/IL23-p40 | Included in IL-2 |
| --- | --- | --- | --- | --- | --- | --- | --- | --- |
| h1 | NSDTR | F | 9 | Healthy | No | NT | Yes | Excluded |
| h2 | NSDTR | F | 4 | Healthy | No | NT | Yes | Yes |
| h3 | NSDTR | F | 3 | Healthy | No | NT | Yes | Yes |
| h4 | NSDTR | F | 8 | Healthy | No | NT | Yes | Yes |
| h5 | NSDTR | F | 4 | Healthy | No | NT | Yes | Yes |
| h6 | NSDTR | F | 7 | Healthy | No | NT | Yes | Yes |
| h7 | NSDTR | F | 4 | Healthy | No | NT | Yes | Yes |
| h8 | NSDTR | F | 4 | Healthy | No | NT | Yes | Yes |
| h9 | NSDTR | F | 5 | Healthy | No | NT | Yes | Yes |
| h10 | NSDTR | F | 8 | Healthy | No | NT | Yes | Yes |
| h11 | NSDTR | M | 9 | Healthy | No | NT | Yes | Yes |
| h12 | NSDTR | M | 6 | Healthy | No | NT | Yes | Yes |
| h13 | NSDTR | M | 3 | Healthy | No | NT | Excluded | Yes |
| h14 | NSDTR | M | 2 | Healthy | No | NT | Yes | Yes |
| h15 | NSDTR | M | 3 | Healthy | No | NT | Yes | Yes |
| h16 | NSDTR | MN | 7 | Healthy | No | NT | Yes | Yes |
| h17 | NSDTR | F | 3 | Healthy | No | NT | No | Yes |
| h18 | NSDTR | M | 3 | Healthy | No | NEG | No | Yes |
| h19 | NSDTR | F | 10 | Healthy | No | NT | No | Yes |
| h20 | NSDTR | M | 1 | Healthy | No | NT | No | Yes |
| h21 | Beagle | MN | 10 | Healthy | No | NT | Yes | No |
| h22 | Beagle | MN | 8 | Healthy | No | NT | Yes | No |
| h23 | Beagle | MN | 8 | Healthy | No | NT | Yes | No |
| h24 | Beagle | F | 6 | Healthy | No | NT | Yes | No |
| h25 | Beagle | F | 6 | Healthy | No | NT | Yes | No |
| h26 | Beagle | F | 6 | Healthy | No | NT | Yes | No |
| h27 | Beagle | F | 6 | Healthy | No | NT | Yes | No |
| h28 | Beagle | F | 6 | Healthy | No | NT | Yes | No |
| h29 | Beagle | F | 6 | Healthy | No | NT | Yes | No |
| h30 | Beagle | M | 5 | Healthy | No | NT | Yes | No |
| h31 | Beagle | M | 3 | Healthy | No | NT | Yes | No |
| h32 | Beagle | M | 3 | Healthy | No | NT | Yes | No |
| H33 | Beagle | F | 3 | Healthy | No | NT | Yes | No |
| h34 | Beagle | F | 3 | Healthy | No | NT | Yes | No |
| h35 | Beagle | F | 3 | Healthy | No | NT | Yes | No |
| h36 | Beagle | F | 3 | Healthy | No | NT | Yes | No |
| I1 | NSDTR | F | 3 | IMRD | 0,5 mg/kg prednisolone EOD | POS | Yes | Yes |
| I2 | NSDTR | F | 4 | IMRD | NSAID | POS | Yes | Yes |
| I3 | NSDTR | F | 6 | IMRD | Untreated | NEG | Yes | Yes |
| I4 | NSDTR | F | 6 | IMRD | 0,3 mg/kg prednisolone EOD | POS | Yes | Yes |
| I5 | NSDTR | F | 5 | IMRD | Untreated | POS | Yes | Yes |
| I6 | NSDTR | F | 1 | IMRD | Untreated | POS | Yes | Yes |
| I7 | NSDTR | F | 4 | IMRD | NSAID | POS | Yes | Yes |
| I8 | NSDTR | F | 4 | IMRD | Untreated | POS | Yes | Yes |
| I9 | NSDTR | F | 9 | IMRD | NSAID | POS | Yes | Yes |
| I10 | NSDTR | M | 9 | IMRD | NSAID | POS | Yes | Yes |
| I11 | NSDTR | M | 3 | IMRD | Untreated | POS | Yes | Yes |
| I12 | NSDTR | M | 5 | IMRD | 0,35 mg/kg | NEG | Yes | Yes |
| I13 | NSDTR | M | 7 | IMRD | 1mg/kg | POS | Yes | Yes |
| I14 | NSDTR | M | 3 | IMRD | 0,5 mg/kg prednisolone EOD | POS | Yes | Yes |
| I15 | NSDTR | M | 7 | IMRD | Untreated | POS | Yes | Yes |
| I16 | NSDTR | M | 2 | IMRD | 2 mg/kg prednisolone | POS | Yes | Yes |
| I17 | NSDTR | MN | 6 | IMRD | Ursodeoxycholic acid | POS | Yes | Yes |
| I18 | NSDTR | M | 3 | IMRD | 0,1mg/kg prednisolone EOD | POS | Yes | Yes |
| I19 | NSDTR | F | 3 | IMRD | Untreated | POS | No | Yes |
| I20 | NSDTR | M | 7 | IMRD | Unknown | POS | No | Yes |
| I21 | NSDTR | F | 3 | IMRD | 1.1 mg/kg prednisolone | POS | No | Yes |
| I22 | NSDTR | F | 5 | IMRD | 0.3 mg/kg prednisolone | POS | No | Yes |
| I23 | NSDTR | F | 5 | IMRD | Untreated | NEG | No | Yes |
| I24 | NSDTR | FN | 6 | IMRD | Untreated | POS | No | Yes |
| I25 | NSDTR | M | 4 | IMRD | Untreated | POS | No | Yes |
| I26 | NSDTR | M | 3 | IMRD | Untreated | POS | No | Yes |
| I27 | NSDTR | MN | 6 | IMRD | NSAID | POS | No | Yes |
| C1 | NSDTR | F | 8 | Lymphoma | Untreated | NT | Yes | Yes |
| C2 | NSDTR | M | 7 | Lymphoma | Untreated | NT | Yes | Yes |
| C3 | NSDTR | F | 12 | Lymphoma | Untreated | NT | Yes | Yes |
| C4 | NSDTR | M | 6 | MCT, subcutaneous | 1 mg/kg prednisolone and vinblastine | NT | Yes | Yes |
| C5 | NSDTR | MN | 8 | MCT, grade 2 | Untreated | NT | Yes | Yes |
| C6 | NSDTR | MN | 12 | MCT, grade 1-2 | Prednisolone | NT | Yes | Yes |
| C7 | NSDTR | FN | 14 | Lymphoma | Untreated | POS | Yes | Yes |
| C8 | NSDTR | F | 7 | MCT, grade 1-2 | Untreated for tumor but levothyroxin for hypothyreosis | NT | Yes | Yes |
| C9 | NSDTR | M | 11 | Lymphoma | Untreated | NT | Yes | Yes |
| C10 | NSDTR | F | 9 | MCT subcutaneous | Untreated | NT | Yes | No |
| C11 | NSDTR | M | 4 | MCT, subcutaneous | Untreated | NT | Yes | Yes |
| C12 | NSDTR | F | 8 | MCT, grade 2 | Tumor excided before sampling but with unclean margins, had radiation | NT | Yes | Yes |
| C13 | NSDTR | F | 6 | Adenocarcinoma | Unknown | NT | No | Yes |
| S1 | NSDTR | M | 1 | SRMA | 1.2 mg/kg prednisolone | NEG | No | Yes |
| S2 | NSDTR | F | 1 | SRMA | Not known | NEG | No | Yes |
| S3 | NSDTR | M | 1 | SRMA | Not known | NEG | No | Yes |
| S4 | NSDTR | M | 1 | SRMA | Prednisolone, dose not known | NEG | No | Yes |
| S5 | NSDTR | M | 1 | SRMA | Untreated | NEG | No | Yes |
| S6 | NSDTR | F | 1 | SRMA | Not known | NEG | No | Yes |
| S7 | NSDTR | M | 1 | SRMA | 1mg/kg prednisolone | NEG | No | Yes |
| S8 | NSDTR | M | 2 | SRMA | 1.5 mg/kg prednisolone | NEG | No | Yes |
| S9 | NSDTR | M | 1 | SRMA | 1.6 mg/kg prednisolone | NEG | No | Yes |

EOD: every other day, F: female, FN; female neutered, IMRD: immune mediated rheumatic disease, M: male, MCT: mast cell tumor, mg/kg: milligrams per kilogram, MN male neutered, NEG.: negative, NSDTR: nova scotia duck tolling retriever, NT: not taken, POS.: positive, SRMA: steroid-responsive meningitis-arteritis
